# Condensin-mediated chromosome organization and gene regulation

**DOI:** 10.3389/fgene.2014.00473

**Published:** 2015-01-13

**Authors:** Alyssa C. Lau, Györgyi Csankovszki

**Affiliations:** Department of Molecular, Cellular and Developmental Biology, University of MichiganAnn Arbor, MI, USA

**Keywords:** *Caenorhabditis elegans*, condensin, dosage compensation, gene expression, chromosome condensation, chromatin, interphase chromosome, epigenetics

## Abstract

In many organisms sexual fate is determined by a chromosome-based method which entails a difference in sex chromosome-linked gene dosage. Consequently, a gene regulatory mechanism called dosage compensation equalizes X-linked gene expression between the sexes. Dosage compensation initiates as cells transition from pluripotency to differentiation. In *Caenorhabditis elegans*, dosage compensation is achieved by the dosage compensation complex (DCC) binding to both X chromosomes in hermaphrodites to downregulate gene expression by twofold. The DCC contains a subcomplex (condensin I^DC^) similar to the evolutionarily conserved condensin complexes which play a fundamental role in chromosome dynamics during mitosis. Therefore, mechanisms related to mitotic chromosome condensation are hypothesized to mediate dosage compensation. Consistent with this hypothesis, monomethylation of histone H4 lysine 20 is increased, whereas acetylation of histone H4 lysine 16 is decreased, both on mitotic chromosomes and on interphase dosage compensated X chromosomes in worms. These observations suggest that interphase dosage compensated X chromosomes maintain some characteristics associated with condensed mitotic chromosome. This chromosome state is stably propagated from one cell generation to the next. In this review we will speculate on how the biochemical activities of condensin can achieve both mitotic chromosome compaction and gene repression.

## INTRODUCTION

Dosage compensation occurs in many species with a difference in sex chromosome number between males (XY or XO) and females (XX). This mechanism equalizes gene expression between the sexes and balances X and autosomal gene expression (Ohno, 1967). Disrupting dosage compensation leads to lethality in the affected sex. Mammals, flies, and worms have distinct dosage compensation strategies. The fly, *Drosophila melanogaster,* upregulates the male X twofold to balance X, and autosomal expression and equalize male to female X-linked gene expression (Conrad and Akhtar, 2011; Ferrari et al., 2014). By contrast, mammals and the nematode, *Caenorhabditis elegans,* are hypothesized to upregulate X chromosome expression in both sexes (Gupta et al., 2006; Lin et al., 2007; Deng et al., 2011, 2013; Lin et al., 2011). This X upregulation balances male X and autosomal expression, but causes X overexpression in females/hermaphrodites. Therefore to compensate for this overexpression, mammals inactivate one X in XX females (Heard and Disteche, 2006; Payer and Lee, 2008; Barakat and Gribnau, 2012), while *C. elegans* downregulates both X chromosomes twofold in the XX hermaphrodites (Csankovszki et al., 2009b; Meyer, 2010).

In *C. elegans*, repression of gene expression is achieved by the dosage compensation complex (DCC), which binds the Xs in hermaphrodites to downregulate gene expression by half. The DCC contains five associated proteins and a subcomplex, condensin I^DC^, which is similar to the evolutionarily conserved condensin complexes that promote chromosome condensation (Csankovszki et al., 2009a). This review focuses on our current understanding of condensins’ biological functions and molecular mechanisms that enable them to achieve both mitotic chromosome compaction and gene repression.

## CONDENSIN COMPLEXES

**FIGURE 1:**
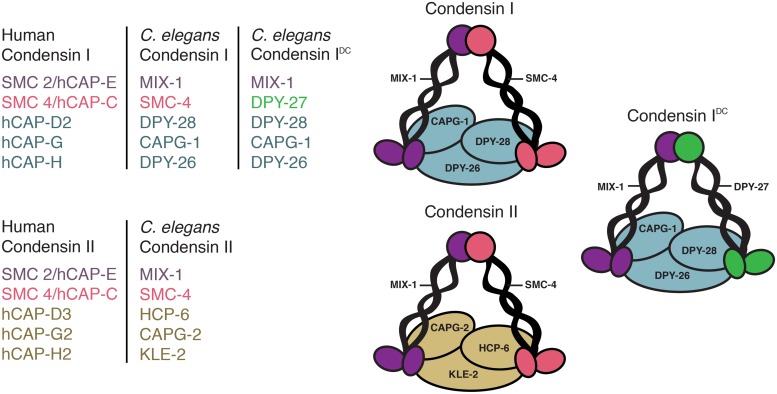
**Three condensin complexes.** *Caenorhabditis elegans* condensin subunits and their human homologs. Condensins I and II share the same pair of MIX-1 and SMC-4 subunits and have three unique chromosome-associated polypeptide (CAP) proteins. Condensin I contains DPY-28, CAPG-1, and DPY-26, while condensin II contains HCP-6, CAPG-2, and KLE-2. In addition, *C. elegans* has a condensin I-like complex (condensin I^DC^) that functions in dosage compensation. Condensin I^DC^ differs from the canonical condensin I by only one subunit: DPY-27 replaces SMC-4.

Condensin complexes are highly conserved five subunit complexes essential for chromosome compaction and segregation in mitosis and meiosis (Hirano, 2012). While yeast has one complex, higher eukaryotes have two, condensins I and II. They consist of a pair of SMC2 and SMC4 subunits belonging to the SMC (structural maintenance of chromosomes) family of chromosomal ATPases and three unique CAP (chromosome-associated polypeptide) proteins. Condensin I contains CAP-D2, CAP-G, and CAP-H, while condensin II contains CAP-D3, CAP-G2, and CAP-H2 (Ono et al., 2003, 2004; Hirota et al., 2004). Uniquely, *C. elegans* has three condensin complexes, condensins I, II, and an additional complex, condensin I^DC^, which contributes exclusively to dosage compensation (Chuang et al., 1994; Lieb et al., 1996, 1998; Tsai et al., 2008; Csankovszki et al., 2009a; **Figure 1**). Interestingly, condensin I^DC^ differs from condensin I complex by only one subunit: DPY-27 replaces SMC-4 (Csankovszki et al., 2009a; Mets and Meyer, 2009). Unlike condensins I and II, which compact and segregate all mitotic and meiotic chromosomes, condensin I^DC^ is X-specific resulting in gene repression in hermaphrodites. Due to the similarity of condensin I and I^DC^, similar mechanisms have long been hypothesized to mediate chromosome compaction and dosage compensation (Chuang et al., 1994). In this review we discuss the mitotic/meiotic and interphase defects caused by condensin mutations or knockdowns. Because condensin is depleted throughout the cell cycle in these experiments, is it is difficult to differentiate between mitotic and interphase functions of condensins. The effects of the activities of condensin in mitosis may persist in interphase and vice versa.

## MITOTIC AND MEIOTIC DEFECTS IN CONDENSIN MUTANTS OR KNOCKDOWNS

In higher eukaryotes condensins I and II have different spatial and temporal localization patterns. Condensin I is cytoplasmic in interphase and accesses chromosomes only after nuclear envelope breakdown (NEBD) in prometaphase, while condensin II is predominantly nuclear and binds chromosomes as soon as condensation begins in prophase (Hirano and Mitchison, 1994; Ono et al., 2004; Gerlich et al., 2006; Collette et al., 2011; Shintomi and Hirano, 2011). This suggests that chromosome condensation may occur in two-steps, first with condensin II in prophase and then with condensin I after NEBD. An exception is mouse embryonic stem cells, where condensin I is nuclear during interphase (Fazzio and Panning, 2010). Furthermore, the global and regional localization of condensins I and II on mitotic chromosomes are different. In monocentric organisms, condensins I and II have non-overlapping distributions within the axis of each sister-chromatid arm, with condensin II enriched at the centromeres (Ono et al., 2003, 2004; Hirota et al., 2004). Similar differences were also found in *C. elegans*, a holocentric organism, in which microtubule attachment sites are scattered throughout the entire length of chromosomes. In *C. elegans*, condensin I associates with mitotic chromosomes in a diffuse discontinuous pattern, while condensin II is enriched at centromeres (Csankovszki et al., 2009a; Collette et al., 2011). Differences in spatial and temporal dynamics of condensins I and II are also present during meiosis (Collette et al., 2011; Lee et al., 2011) Recent studies explored the genome-wide distribution of condensin complexes at high resolution. These studies have uncovered both unique and similar binding sites of condensins I and II (Kim et al., 2013; Kranz et al., 2013; Van Bortle et al., 2014).

Although the two mitotic condensins are structurally similar, this difference in localization suggests that they may play distinct roles in chromosome organization. Consistent with this idea, the depletion of condensin I or II alone results in distinct chromosomal defects, while the depletion of both condensins leads to more severe defects (Ono et al., 2003, 2004; Hirota et al., 2004). Condensin I facilitates lateral compaction of mitotic chromosomes, whereas condensin II primarily contributes to axial compaction (Ono et al., 2003; Hirota et al., 2004; Shintomi and Hirano, 2011; Green et al., 2012). The roles the two condensins play in mitosis varies among different eukaryotic species. For example, in *Xenopus laevis*, *S. pombe,* and *S. cerevisiae*, condensin is required for mitotic chromosome condensation and mechanical stability (Hirano and Mitchison, 1994; Hirano et al., 1997; Sutani et al., 1999; Freeman et al., 2000; Gerlich et al., 2006). Condensins also play critical roles in meiotic chromosome compaction and segregation (Chan et al., 2004; Lee et al., 2011). During *C. elegans* meiosis, depletion of condensin I or II leads to an expansion of chromosome axis (Mets and Meyer, 2009). A study using *Xenopus laevis* egg extracts showed that a critical determinant of chromatid shape is the relative ratio of condensins I and II (Shintomi and Hirano, 2011). In other organisms, such as mammals and worms, condensin II plays a primary role in prophase condensation (Hagstrom et al., 2002; Hirota et al., 2004; Csankovszki et al., 2009a). Interestingly, when both condensins are depleted in *Drosophila*, worms, mammals, and chicken DT40 cells, the primary defect appears to be anaphase chromatin bridging, rather than chromosome condensation (Hirano, 2012). This suggests that other factors may contribute to mitotic chromosome condensation in addition to condensin.

## INTERPHASE DEFECTS IN CONDENSIN MUTANTS OR KNOCKDOWNS

Emerging evidence suggests that condensin complexes also contribute to a variety of interphase functions. It is believed that condensin II, rather than condensin I, plays a primary role in interphase, since in condensin II is nuclear throughout the cell cycle, while condensin I is cytoplasmic in interphase (Hirota et al., 2004; Ono et al., 2004; Gerlich et al., 2006; Collette et al., 2011; Shintomi and Hirano, 2011). In *Drosophila* ovarian nurse cells, condensin II disassembles polytene chromosomes into unpaired homologous chromosomes. This unpairing activity leads to interphase chromosome compaction (Hartl et al., 2008a,b; Joyce et al., 2012). In *Drosophila* cell lines, condensin-mediated interphase condensation is normally limited by the SCF^Slimb^ ubiquitin ligase. The condensin II subunit CAP-H2 is a Slimb target for ubiquitin-mediated degradation. Degradation of CAP-H2 inactivates condensin II, thereby preventing interphase chromatin reorganization. Inhibition of SCF^Slimb^ leads to CAP-H2 stabilization, resulting in chromosome unpairing and nuclear structural abnormalities (Buster et al., 2013). This suggests that in interphase, condensin II activity is suppressed in order to prevent chromosome condensation and changes in nuclear organization. In addition, condensin II also regulates chromosome territory formation in multiple cell types. This conclusion is based on the finding that CAP-H2 promotes axial compaction and proper compartmentalization of the interphase nucleus into chromosome territories in both nurse cells and salivary glands (Bauer et al., 2012). These findings suggest that the interphase function of condensin II is similar to its role in axial compaction of mitotic chromosomes.

Condensin subunits also play a role in regulation of cell-type specific gene expression. In mice, chromosome compaction by condensin II is required for T-cell development and maintenance of the quiescent state. Mutations in the condensin II subunit kleisin-β (CAP-H2) lead to open chromatin configuration and upregulation of normally silenced genes. After T-cell activation, chromatin decondenses and transcription is upregulated (Rawlings et al., 2011). Similarly, murine CAP-G2 represses transcription during erythroid cell differentiation. During erythroid cell maturation nuclei gradually condense, mediated by condensin (Xu et al., 2006). Condensin is also required for higher-order chromatin compaction and viability in ES cells. (Fazzio and Panning, 2010).

Yeast condensin has also been shown to play a role in interphase chromatin organization and RNA polymerase III-transcribed gene clustering. In budding and fission yeast, the three-dimensional organization of the genome is facilitated in part by condensin-mediated localization of RNA-polymerase III genes within the nucleus (Iwasaki et al., 2010). In budding yeast, tRNA genes are clustered at the nucleolus in a condensin-dependent manner. Mutations in yeast condensin subunits cause tRNA gene positioning defects and partially inhibit tRNA gene-mediated silencing (Haeusler et al., 2008), illustrating another connection between condensin-mediated genome organization and gene expression.

In the above examples, condensin either affected the entire genome, or a subset of genes scattered on different chromosomes. By contrast, in *C. elegans*, condensin I^DC^ causes chromosome-specific changes. Consistent with a role in chromosome condensation, *C. elegans* condensin I^DC^ mediates compaction of dosage compensated X chromosomes in interphase. Condensin I^DC^-bound X chromosomes are more compact than expected by DNA content, whereas mutations or depletions of condensin I^DC^ result in decompaction of X chromosome territories (Lau et al., 2014). These results are consistent with the model that reduction of X-linked gene expression occurs as a result of condensin I^DC^-mediated changes in chromatin structure. However, whether this condensation is a cause or consequence of transcriptional repression is unknown.

## CONDENSIN AND CHROMATIN MEDIATED CHROMOSOME COMPACTION

**FIGURE 2:**
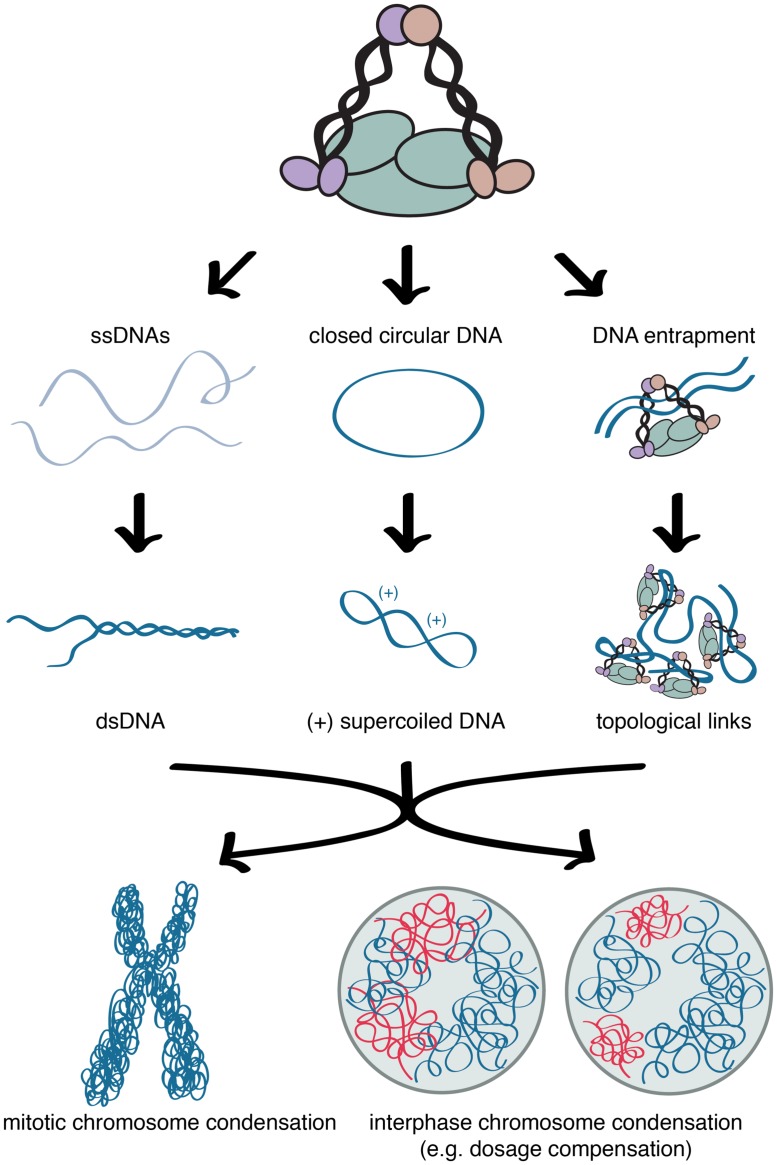
**Condensin and chromatin mediated chromosome compaction.** Similar distributions of histone modifications and condensin in condensed mitotic chromosomes and interphase dosage compensated X chromosomes. Compaction is accompanied by enrichment of H4K20me1 and depletion of H4K16ac in both mitotic chromosome condensation and interphase dosage compensated X chromosomes.

In addition to condensin-mediated condensation, histone modifications also influence chromatin compaction during mitosis and the structure of *C. elegans* dosage compensated X chromosomes. The similarity of chromatin modifications between mitotic chromosome and dosage compensated X chromosomes of *C. elegans* is consistent with X chromosome repression being mediated by mechanisms similar to mitotic chromosome condensation. On both mitotic chromosomes and interphase dosage compensated X chromosomes monomethylation of histone H4 lysine 20 (H4K20) is increased whereas acetylation of histone H4 lysine 16 (H4K16) is decreased (**Figure 2**; Rice et al., 2002; Oda et al., 2009; Vielle et al., 2012; Wells et al., 2012; Wilkins et al., 2014). During cell cycle progression the levels of both the H4K20 methyltransferase, PR-SET-7, and H4K20me1 increase in G2, remain high in mitosis, and decrease in G1 (Rice et al., 2002; Oda et al., 2009). Additionally, the depletion of PR-SET-7 leads to cell cycle defects, and mitotic and interphase chromosome decondensation (Oda et al., 2009), illustrating the importance of H4K20me1 in mitosis and chromosome compaction. By contrast, H4K16ac levels increase during S phase and decrease during mitosis (Rice et al., 2002; Wilkins et al., 2014). This data is consistent with findings that H4K20me1 antagonizes H4K16ac (Nishioka et al., 2002). In yeast, H4K16ac deacetylation in mitosis is achieved by Hst2 (Sir2 homolog), which is recruited by histone H3 phosphorylated on serine 10 (Wilkins et al., 2014). Deacetylation of H4K16ac leads to stronger interactions between H2A and H4 on neighboring nucleosomes, leading higher degree of condensation (Shogren-Knaak et al., 2006; Wilkins et al., 2014). In mitosis, this cascade of histone modifications is proposed to drive chromatin hypercondensation, independently from condensin (Wilkins et al., 2014). However, it has been shown that mitotic condensin II subunits CAP-D3 and CAP-G2 are capable of binding H4K20me1, suggesting H4K20me1 may play a role in condensin II loading (Liu et al., 2010).

*Caenorhabditis elegans* interphase dosage compensated X chromosomes show similar changes in histone modifications: H4K20me1 is increased, whereas H4K16ac is decreased on X. The enrichment of H4K20me1 is regulated not only by the DCC but also the H4K20 monomethylase, SET-1 (PR-SET7 homolog), and the H4K20 di- and trimethylase, SET-4 (SUV4-20 homolog; Vielle et al., 2012; Wells et al., 2012). The DCC also regulates SIR-2.1 (Sir2 homolog), which mediates the depletion of H4K16ac on X chromosomes (Wells et al., 2012). This cascade of histone modifications drives X chromatin condensation in a DCC- (therefore condensin-) dependent manner (Lau et al., 2014). By contrast, in mitosis, these histone modifications are proposed to act independently of condensin (Wilkins et al., 2014). These observations suggest that interphase dosage compensated X chromosomes maintain some characteristics associated with condensed mitotic chromosome.

## MOLECULAR MECHANISMS OF CONDENSIN ACTIVITY

The mechanisms by which condensin generates and maintains chromosome condensation in interphase and mitosis are highly debated and poorly understood. The biochemical mechanisms discussed below have been proposed to contribute to chromosome condensation. However, whether these activities contribute to condensin’s interphase or mitotic functions, or both, is unknown.

**FIGURE 3:**
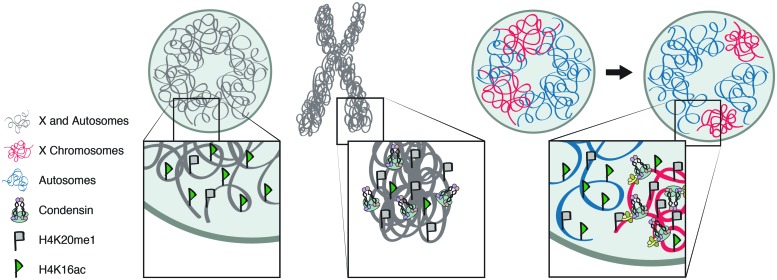
**Molecular mechanisms of condensin activity.** The proposed mechanisms by which condensin generates and maintains chromosome condensation in interphase and mitosis. Condensin’s structural maintenance of chromosomes (SMC) proteins can reanneal complementary ssDNAs into dsDNAs, in preparation for subsequent coiling steps. Condensin can also introduce ATP-dependent positive supercoils into DNA *in vitro*. Alternatively, or in addition, condensin is proposed to entrap the chromatin fibers in its ring-like structure.

The two SMC proteins of condensin are able to hydrolyze ATP and this activity is believed to be essential for regulating higher-order chromatin structure (Kimura and Hirano, 1997; Hirano, 2012). The SMC proteins also have the ability to reanneal complementary ssDNAs into dsDNAs (Sakai et al., 2003), perhaps as a preparatory step for the formation of mitotic chromosomes (**Figure 3**). The best-characterized mechanism of mitotic condensin, detected in many eukaryotic species is the ability to introduce ATP-dependent positive supercoils into DNA *in vitro* (Kimura and Hirano, 1997, 2000; Kimura et al., 2001; Hagstrom et al., 2002; St-Pierre et al., 2009). Using closed circular DNA and in the presence of topoisomerase I, mitotic condensin I is able to supercoil the DNA with its DNA-stimulated ATPase activity (Kimura and Hirano, 1997). This activity requires the entire five-unit complex. The SMC proteins alone do not have ATPase activity and cannot bind chromatin *in vitro* (Kimura and Hirano, 2000). Positive supercoiling is proposed to facilitate topoisomerase II-mediated decatenation of the sister chromatids and lead to the formation of chiral loops. Higher order assemblies by condensin–condensin interactions can then compact the chromatin fiber (**Figure 3**; Kimura et al., 1999; Baxter et al., 2011).

Phosphorylation of condensin’s CAP subunits by the kinase CDK1 (cyclin-dependent kinase 1) is required to supercoil DNA and initiate mitotic chromosome condensation *in vitro* (Kimura et al., 1998; Takemoto et al., 2006). By contrast, the supercoiling activity is not detected when the interphase form of condensin is incubated with circular DNA in the presence of ATP and topoisomerase I (Kimura et al., 1998). In fact, phosphorylation of condensin I by a different kinase, CK2 (casein kinase 2), suppresses supercoiling activity during interphase (Takemoto et al., 2006). This suggests that condensin I-mediated DNA supercoiling may not be involved in chromosome compaction during interphase. However, it is not known whether condensin II-mediated supercoiling or additional molecular mechanisms drive interphase chromatin organization.

Alternatively, or in addition to supercoiling, condensin is proposed to entrap the chromatin fibers in a ring-like structure (Cuylen et al., 2011). This hypothesis is based on condensin’s resemblance to cohesin, both containing a pair of SMC proteins, forming a V-shape, and additional non-SMC proteins, proposed to close the ring (**Figure 3**). Cohesin is believed to hold pairs of the sister chromatids together by entrapping DNA from each chromatid within its ring-like structure (Haering et al., 2008). A recent study on yeast minichromosomes provided evidence that condensin forms similar topological links by encircling DNA. Linearization of the minichromosome DNA or opening the condensin ring eliminated the association between the DNA and condensin (Cuylen et al., 2011). While cohesion is believed to hold sister chromatids together, condensin is proposed to entrap different sections of the same DNA molecule, to facilitate condensation.

Condensin’s ability to shape chromosomes is further illustrated by its localization to topologically associating domain (TAD) boundaries in interphase chromosomes. A TAD is a contiguous chromosomal region with high frequency of interactions between sequences within the TAD, but few interactions with sequences outside the TAD. In interphase *Drosophila,* mouse, and human ES cells, condensin II has been found to localize at high occupancy architectural protein binding sites (APBSs) located at the borders of TADs (Van Bortle et al., 2014). Localization of condensin II at TAD boundaries, together with its ability to entrap DNA, suggests a possible mechanisms for regulating interphase chromatin organization. Unlike interphase chromatin, which is partitioned into small sub-megabase TADs and large multi-megabase compartments (Dekker and Mirny, 2013), mitotic chromosomes do not exhibit chromosome compartments and TADs (Naumova et al., 2013). Instead it is believed that chromatin is linearly compacted into consecutive loops, potentially by SMC complexes, and then homogeneous axial compression leads to the formation of dense mitotic chromosomes (Naumova et al., 2013). Thus, there may be unique and overlapping mechanisms involved in condensin-mediated chromosome compaction in interphase and mitosis.

Which of these biochemical activities, if any, contribute to *C. elegans* dosage compensation is unknown. Mutations in the ATPase domains of DPY-27 and MIX-1 lead to dosage compensation defects (Chuang et al., 1994; Lieb et al., 1998), suggesting that the ATPase activity is required for the mechanisms that mediate dosage compensation. Whether condensin I^DC^ is able to reanneal single stranded DNA, supercoil DNA, or entrap chromatin fibers has not been investigated. Future studies of condensin’s biochemical activities will reveal how condensin is able to achieve both mitotic chromosome compaction and gene repression.

## CONCLUSION

Condensin complexes emerged as important regulators of chromatin organization throughout the cell cycle. Recent studies revealed that in addition to their role in mitotic chromosome condensation and segregation, condensins function in diverse interphase processes. Emerging evidence connects mitotic condensin-mediated condensation with epigenetic control of gene expression. Although there is increasing understanding of the biological functions of condensins in mitosis, meiosis, and interphase, the molecular mechanisms of condensin activity are still poorly understood. Since most of our knowledge of these molecular mechanisms comes from analysis of condensin I in mitosis, it will be important to examine the mechanistic similarities and differences between the activities of condensins I and II, both in mitosis and interphase. Studying *C. elegans* condensin I^DC^’s function in dosage compensation will shed further light on how condensin affects interphase chromosome organization and how the activities involved differ from condensin’s function in mitosis.

## Conflict of Interest Statement

The authors declare that the research was conducted in the absence of any commercial or financial relationships that could be construed as a potential conflict of interest.
